# Smart Healing for Wound Repair: Emerging Multifunctional Strategies in Personalized Regenerative Medicine and Their Relevance to Orthopedics

**DOI:** 10.3390/antibiotics15010036

**Published:** 2026-01-01

**Authors:** Carla Renata Arciola, Veronica Panichi, Gloria Bua, Silvia Costantini, Giulia Bottau, Stefano Ravaioli, Eleonora Capponi, Davide Campoccia

**Affiliations:** 1 Department of Medical and Surgical Sciences (DIMEC), University of Bologna, Via San Giacomo 14, 40126 Bologna, Italy; silvia.costantini6@unibo.it; 2 Laboratory of Immunorheumatology and Tissue Regeneration, Laboratory on Pathology of Implant Infections, IRCCS Istituto Ortopedico Rizzoli, Via di Barbiano 1/10, 40136 Bologna, Italy; 3 Laboratory of Immunorheumatology and Tissue Regeneration, IRCCS Istituto Ortopedico Rizzoli, Via di Barbiano 1/10, 40136 Bologna, Italy; veronica.panichi@ior.it; 4 Laboratory on Pathology of Implant Infections, IRCCS Istituto Ortopedico Rizzoli, Via di Barbiano 1/10, 40136 Bologna, Italy; gloria.bua@ior.it (G.B.); giulia.bottau@ior.it (G.B.); stefano.ravaioli@ior.it (S.R.); eleonora.capponi@ior.it (E.C.)

**Keywords:** wound healing, wounds, chronic, antibacterial wound dressing, anti-bacterial/antibiofilm strategies, regenerative medicine, personalized medicine, functional biomaterials, antibacterial hydrogels, nanomaterials, stimuli-responsive materials

## Abstract

To address the challenges in wound healing, clinical management increasingly demands targeted, adaptive, responsive, and patient-centered strategies. This is especially true for wounds characterized by delayed healing and a high risk of infection. Advances in regenerative medicine and biomaterial technologies are fostering the development of multifunctional approaches that integrate tissue regeneration, antibacterial/antibiofilm activity, immunomodulation, and real-time monitoring. This paper surveys emerging platforms, including both natural and synthetic scaffolds, hydrogels enriched with platelet-derived growth factors, glycosaminoglycan mimetics, bioactive peptides (such as GHK-Cu and antimicrobial peptides), nanoscaffolds, and stimuli-responsive systems. The paper also explores cutting-edge technologies such as water-powered, electronics-free dressings that deliver localized electrical stimulation; biodegradable bioelectric sutures that produce self-sustained mechano-electrical signals; and sensory bandages that monitor pH, moisture, temperature, and bacterial contamination in real-time while enabling on-demand drug release with pro-regenerative, antibacterial, and other therapeutic functionalities. Further therapeutic approaches include natural matrices, exosomes, gene editing, 3D bioprinting, and AI-assisted design. Particular attention is paid to orthopedic applications and orthopedic implant infection. A brief section addresses the still unresolved challenge of articular cartilage regeneration. Interdisciplinary innovation, integrating insights from molecular biology through engineering, plays a central role in translating novel strategies into tailored, clinically effective wound management solutions.

## 1. Introduction

Wound healing remains a significant challenge in clinical practice and scientific research, with serious consequences for patients and healthcare systems. The global burden of chronic wounds is estimated at a prevalence of 1 and 2% [1], although recent disease-specific epidemiological data remain limited. Wounds may be classified as acute or chronic, the latter defined as those that do not heal in a normal and timely manner, typically within three months, or that progress through the repair process but fail to achieve lasting structural and functional recovery [2]. These lesions may evolve into complex, difficult-to-manage conditions, particularly in surgical settings such as orthopedic implantology and in patients with underlying comorbidities [3,4].

Among these comorbidities, diabetes mellitus, obesity, nutritional deficiencies, autoimmune diseases, and peripheral vascular disorders are common factors that impair tissue repair, most notably in the context of chronic wounds. Among local factors disrupting homeostasis, hence the healing process, reactive oxygen species (ROS) are major contributors to cellular damage and inflammation, emphasizing their central role in the tissue pathology associated with wound healing [4].

In particular, diabetes mellitus is characterized by both macroangiopathy and microangiopathy, peripheral neuropathies (often presenting as hypoesthesia that reduces sensitivity to minor trauma), hampered neutrophil and macrophage phagocytosis, and disturbed extracellular matrix remodeling, all of which are pathogenetically linked to protein glycation processes triggered by hyperglycemia [5,6].

Obesity negatively impacts wound healing through a combination of chronic low-grade systemic inflammation and tissue hypoperfusion [7,8,9].

Nutritional deficiencies, common in the elderly, particularly protein and vitamin deficiencies, also represent key risk factors for delayed wound healing [10]. For instance, vitamin C acts as a cofactor for enzymes essential for collagen stabilization and effective healing [11].

Autoimmune disorders, such as rheumatoid arthritis, are characterized by chronic systemic inflammation, which interferes with wound healing dynamics [12]. Furthermore, disease-modifying antirheumatic drugs (DMARDs) and corticosteroids used in treatment may perturb tissue repair mechanisms.

Peripheral arterial disease reduces tissue perfusion and oxygenation, leading to delayed granulation tissue formation, and an increased risk of necrosis and infection [13]. Chronic venous insufficiency hinders wound healing through blood stasis, poor oxygenation, and inflammation, and is associated with edema that is predominantly transudative due to venous hypertension, although it may acquire exudative features in advanced stages characterized by chronic inflammation [14].

Although wound healing is often preserved in cancer patients, the oncological disease process and treatments such as chemotherapy and radiotherapy can weaken immune responses and increase the risk of wound dehiscence and infection, especially in the perioperative setting [15].

In this paper, the physiological process of wound healing is initially delineated, highlighting its distinct phases and key molecular mediators. The glance then turns to the major unresolved challenges in orthopedics, concisely contextualizing the pertinent clinical scenarios.

Building on this foundation, an updated and comprehensive overview is provided of recent advances in biomaterials, regenerative medicine, and therapeutic platforms aimed at enhancing tissue repair and controlling infection. Collectively, these developments foster the pursuit of effective, versatile, and personalized approaches to wound management. The paper concludes with reflections on current limitations and future directions.

## 2. Molecular Complexity of Regeneration and Repair

Tissue regeneration and repair are highly dynamic and orchestrated processes involving various cell types, molecular mediators, and a multiplicity of microenvironmental signals and reverberations. At its core, regeneration and repair integrate inflammatory responses, cellular metabolism, immune cell plasticity, extracellular matrix remodeling, and stem/progenitor cell activation to restore tissue structure and function.

As highlighted by Eming et al., the interplay between inflammation and the metabolic reprogramming of immune and stromal cells guides the regenerative trajectory of injured tissues, emphasizing that inflammation is not merely a transient defense mechanism but rather an essential regulator of healing and repair processes [16].

**Figure 1:**
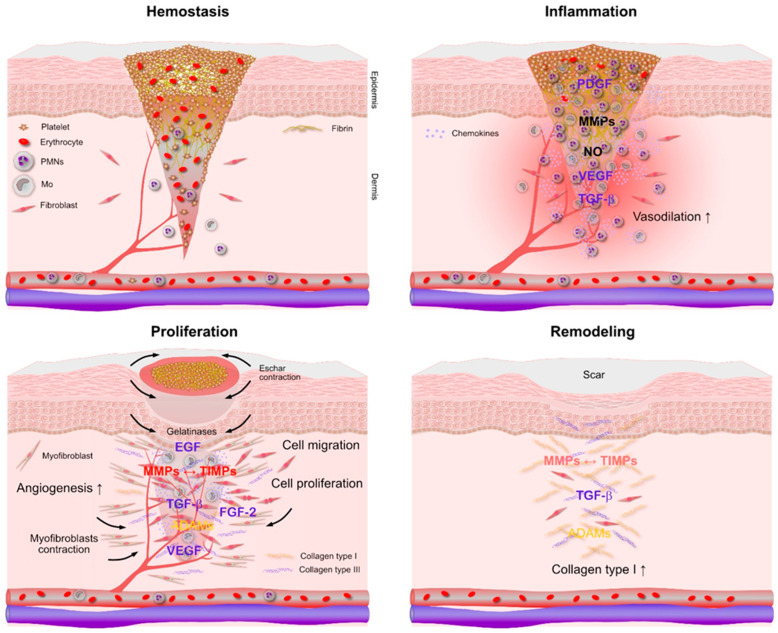
A schematic overview of the dynamic and overlapping phases of wound healing: hemostasis, inflammation, proliferation, and remodeling. Key cellular actors (platelets, macrophages, fibroblasts, keratinocytes, and endothelial cells) and representative molecular mediators (e.g., IL-6, VEGF, PDGF, TGF-β, BMPs) are shown. Emphasis turns to the coordinated roles of immune and stromal cells in supporting tissue repair and regeneration. The failure to properly transition between phases, especially in the presence of infection, implants, or systemic comorbidities, may result in chronic non-healing wounds or fibrosis.

The importance of inflammation clearly emerges when analyzing the complexity of the wound healing process. The normal tissue healing process is classically described to consist of four distinct phases, which are illustrated in Figure 1.

The first phase of healing takes place as soon as the bleeding originates from broken capillaries and blood vessels. This phase, known as “hemostasis”, is characterized by the immediate (minutes to hours) activation and clustering of platelets that come into contact with the collagen of the extracellular matrix and the concomitant activation of the coagulation cascade. A clot consisting of packed platelets and fibrin is generated that stops the bleeding and seals the broken vessels [17].

The subsequent “inflammation” phase is characterized by the progressive migration of leukocytes, polymorphonuclear cells, in first place, subsequently followed by monocytes. This second phase is usually between 3 to 5 days depending on the efficiency of the organism response to debris clean-up. Hypoxic conditions and nitrogen monoxide (NO) produced by activated PMNs cause vasodilation, which in turn increases the leukocytes’ local influx. During this phase, pro-inflammatory chemokines such as interleukin-1β (IL-1β), tumor necrosis factor-alpha (TNF-α) and IL-6 are released by activated leukocytes generating acute inflammation. Matrix metalloproteinases (MMPs) such as MMP-8 are expressed by PMNs [18] and help to recruit further leukocytes to the necrotic area. A series of growth factors are produced during this phase including: platelet-derived growth factor (PDGF), which stimulates proliferation, migration and differentiation of endothelial and fibroblast cells; vascular endothelial growth factor (VEGF), which stimulates the formation of new blood vessels and capillaries by promoting proliferation and migration of endothelial cells; and transforming growth factor-β (TGF-β). Bearing pleiotropic effects, TGF-β is a powerful chemoattractant for fibroblasts and promotes the fibrotic response, i.e., an increased collagen expression by fibroblasts necessary for tissue repair but, importantly, leading to pathological outcomes if unbalanced [19]. It also supports wound closure favoring keratinocyte migration and re-epithelialization.

The next phase, the “proliferation” phase, originates from the impulse to cell migration and proliferation determined by the consistent production of growth factors during inflammation. This critical phase, lasting up to 3 weeks, involves the transition from acute inflammation to regenerative inflammation, with profound implication in wound repair and healing. Monocyte-derived macrophages assume an anti-inflammatory, pro-regenerative phenotype, differing from the pro-inflammatory phenotype of the inflammation phase. New anti-inflammatory cytokines are highly expressed by pro-regenerative macrophages and characterize the transition to the proliferation phase (e.g., IL-10). Angiogenesis promoted by growth factors leads to neovascularization and granulation tissue formation. Simultaneously, fibroblasts and myofibroblasts are recruited and proliferate. Locally produced by macrophages, fibroblast growth factor-2 (FGF-2) and TGF-β stimulate abundant collagen expression. During the proliferation phase, type III collagen prevails over type I collagen and forms a temporary matrix. While eschar shrinkage progressively takes place by dehydration, myofibroblasts determine a contraction of the underlying healing tissue. MMPs, including gelatinases and the ADAMs family of enzymes (an acronym for A *Disintegrin* And *Metalloproteinases*), proteolytically cleave proteins of the extracellular matrix as well as cell receptors and signaling molecules.

Other endogenous regulators, named tissue inhibitors of metalloproteinases (TIMPs), modulate and prevent unbalanced activity of MMPs [20]. Epidermal growth factor (EGF) acts by stimulating the migration and proliferation of keratinocytes, fibroblasts and myofibroblasts. It helps to re-establish the skin barrier functions by enhancing re-epithelialization.

The latest “remodeling” phase involves scar formation and restoration of a normal tissue structure. The timeframe for this process is the most variable, extending from weeks to years and is therefore crucial, especially in chronic wounds. The extracellular matrix is remodeled with type II collagen becoming dominant and forming thicker parallel fibers, conferring greater strength to the final healed tissue. Each individual mechanism involved is strictly regulated and must be finely tuned to ensure proper tissue regeneration and prevent pathological outcomes.

Among immune cells, macrophages play a pivotal role as molecular orchestrators of regeneration and repair. Their plasticity allows them to shift from pro-inflammatory (M1-like) to pro-regenerative (M2-like) phenotypes in response to local cues.

Recent studies, including the work by Wang et al., have further defined their role as biosensors and effectors of microenvironmental changes, capable of modulating angiogenesis, matrix remodeling, and progenitor cell activation through a wide array of cytokines, growth factors, and metabolic signals [21].

## 3. Clinical Challenges in Orthopedics

Surgical wounds, particularly in orthopedic implantology, pose unique challenges due to the high risk of infection and complex tissue damage [3]. Rising antimicrobial resistance amplifies the threat of chronic infections [22], a danger made all the more uncertain by the absence of up-to-date global epidemiological data.

The Centers for Disease Control and Prevention (CDC) surgical wound classification (SWC) system categorizes wounds into four classes (clean, clean/contaminated, contaminated, and dirty) based on infection risk. However, this system may not fully capture the complexities of orthopedic and implant-associated wounds, where even minimal contamination can severely impact outcomes [23].

In elective orthopedic surgery, most procedures fall within Class I (clean), but open fractures or pre-existing infections often shift classification toward more severe categories (Class III or IV). To address this, more specific classifications such as the Gustilo–Anderson system for open fractures have been developed, taking into account laceration size, soft tissue injury, contamination, and vascular damage, guiding treatment and predicting infection risk [24,25]. The heterogeneity of orthopedic wound classifications complicates the selection of the most appropriate therapeutic strategy, as treatment must be carefully tailored to the severity and specific characteristics of each wound.

Further hindering the therapeutic approach, patient-specific other factors complicate healing in orthopedic contexts when occurring in relation to tissue degeneration and “diabesity” (the coexistence of obesity and type 2 diabetes mellitus) [26,27,28]. The presence of implants and biomaterials adds another layer of complexity. Through various mechanisms such as local microtraumas, debris release, and frustration of neutrophil phagocytosis, these materials can induce local mechanical and inflammatory stress, promote bacterial adhesion, and encourage biofilm formation, all of which may hinder healing [3]. In such cases, wounds frequently exhibit persistent inflammation [29] and undermined tissue regeneration. The convergence of these complex challenges renders the development of novel strategies exceptionally difficult, as it requires careful consideration of a multitude of interdependent factors.

Recent advances in biomaterial science are opening new avenues for the development of multifunctional platforms that could successfully address this vast range of limitations. These innovations are designed not only to mitigate complications but also to actively support tissue regeneration based on the wound severity but also prevent microbial contamination and biofilm formation while modulating inflammatory responses.

Another major clinical issue in orthopedics is the management of diabetic foot ulcers. Diabetic patients are more prone to infection, partly due to advanced glycation end products (AGEs), which alter immune cell receptors and subvert their immune function. Consequently, wounds often become chronic, refractory to natural healing, and necessitating targeted advanced interventions [30].

In response, multifunctional biomaterials, smart devices, and bioactive nanostructures are increasingly proposed as promising tools to promote tissue regeneration, modulate inflammation, and counteract infection, offering new prospects for integrated and personalized management of chronic wounds.

Recent progress in understanding the cellular and molecular mechanisms of tissue repair, including intracellular signaling, immune cell crosstalk, and extracellular matrix remodeling, has paved the way for translational approaches bridging bench and bedside. Furthermore, contributions from physics and chemistry have introduced smart, stimuli-responsive materials capable of interacting dynamically with the wound microenvironment by responding to changes in temperature, pH, or pathogen presence.

Hence, there is a growing need for multifunctional therapeutic systems capable of simultaneously promoting regeneration, controlling inflammation, and preventing infection. Ideal materials should enhance cell proliferation, facilitate re-epithelialization, reduce or turn off chronic immune activation, and inhibit bacterial colonization and biofilm formation.

To meet these goals, various biomaterials and bioactive molecules are being extensively investigated, including natural biopolymers (e.g., chitosan, alginate, collagen), synthetic hydrogels, peptide-based agents, nanocomposite scaffolds, and hybrid materials with controlled or stimuli-responsive release properties.

**Figure 2:**
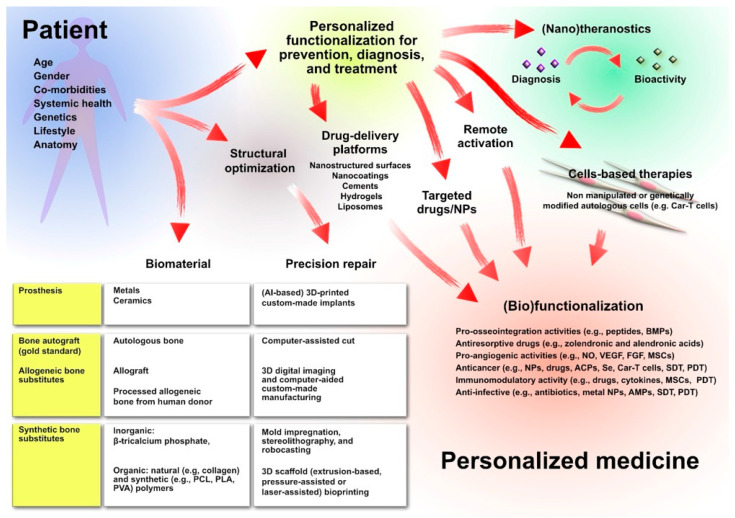
Current and future perspectives for personalized medicine in orthopedics, from precise custom-made 3D manufacturing of the implants to futuristic customized multifunctionalization of the implant by new advancing technologies.

Figure 2 illustrates that personalized medicine in orthopedics is already taking place as far as the choice of biomaterial and precision repair are concerned, but further new possibilities will be probably accessible in the future, offering multifunctional solutions that address or prevent different concomitant pathologies, modulate the tissue response, and determine a faster and improved osteointegration of the implants.

## 4. Multifunctional Scaffold-Based Therapeutic Strategies for Orthopedics Wound Healing

### 4.1. Chitosan as a Multifunctional Platform for Wound Healing

Chitosan, a biomaterial with decades of application history, continues to play a central role in current regenerative and therapeutic strategies. This polysaccharide, derived from the deacetylation of chitin, stands out as an extremely versatile material in the field of tissue regeneration. Biocompatible, biodegradable, and non-toxic, it exhibits significant antimicrobial activity, tissue adhesion capabilities, and modulation of the inflammatory response, making it particularly suitable for the treatment of both acute and chronic wounds [31].

The structural properties of chitosan, such as molecular weight and degree of deacetylation, directly influence its biological and mechanical performance. A higher degree of deacetylation values enhances cellular adhesion and proliferation, whereas lower molecular weights promote biodegradability. Combining chitosan with other natural or synthetic polymers (e.g., hyaluronic acid, collagen, or PVA), or with nanoparticles (metal oxides, titanium dioxide, etc.) further expands its clinical potential [32,33].

Specifically, chitosan-based films and hydrogels are well-suited for the controlled release of therapeutic agents, including growth factors, antibiotics, metal ions, antimicrobial peptides, and even extracellular vesicles such as exosomes. To enhance mechanical properties and stability, physical or chemical crosslinkers and photoinitiated crosslinking strategies can be employed. For example, the incorporation of additives like titanium dioxide (TiO_2_) improves the mechanical strength and photocatalytic activity of the system [34,35].

Clinical application of chitosan is further enhanced by nanobiotechnological approaches. Recently, Wang et al. [36] developed a multifunctional biomimetic hydrogel based on quaternized chitosan and manganese dioxide nanoparticles coated with polydopamine. This system mimics the enzymatic activities of superoxide dismutase and catalase, reducing reactive oxygen species (ROS) accumulation and generating oxygen within the hypoxic microenvironment of diabetic wounds. Moreover, the hydrogel modulates macrophage polarization toward anti-inflammatory M2 phenotypes, exerts antibacterial effects against *Staphylococcus aureus*, and promotes neovascularization and dermal collagen synthesis [36]. In another approach, Yu et al. [37] developed a thermosensitive hydrogel containing zinc oxide nanoparticles functionalized with exosomes derived from human endothelial cells. Furthermore, polymer matrices composed of chitosan, β-glycerophosphate, and genipin enable the controlled release of extracellular vesicles, which synergistically exert anti-inflammatory, angiogenic, and antibacterial effects, significantly enhancing the healing of infected wounds [28,29,30,31,32].

These formulations demonstrate that chitosan serves not only as a physical support matrix but also as an active biochemical modulator capable of integrating with advanced therapeutic strategies. Its multifunctional nature makes it a versatile platform to address the complexity of chronic wounds, especially in diabetic patients or those with vasculopathies. Furthermore, due to its antimicrobial properties and ability to support tissue regeneration, chitosan-based materials lend themselves to managing surgical wounds in orthopedic and implant-related procedures, where infection control and enhanced soft tissue healing are critical for implant success and limb preservation.

Important applications of chitosan are associated with its versatility in the production of degradable layer-by-layer films and nano-coatings, which are suitable platforms for surface functionalization of the implants and drug delivery [38,39].

### 4.2. Ovine Forestomach Matrix (OFM): An Advanced Regenerative Approach for Chronic Wounds and Reconstructive Surgery

Advanced tissue regeneration increasingly relies on decellularized extracellular matrices (ECMs) as biological scaffolds capable of providing a three-dimensional environment favorable to cell migration, proliferation, and differentiation.

Ovine forestomach matrix (OFM) is a bioscaffold composed of decellularized extracellular matrix (ECM) that has been proposed as a dermal matrix in various soft-tissue reconstruction procedures. This decellularized matrix retains the structural and bioactive components of the native ECM, including collagen, glycosaminoglycans, and basement membrane proteins, providing a microenvironment conducive to tissue regeneration and functional tissue formation, with minimal immunogenicity. In a very recent retrospective study, Aburn et al. [40] compared the performance of OFM with that of a collagen/oxidized regenerated cellulose (collagen/ORC) matrix in the treatment of venous leg ulcers. The results showed faster wound closure and higher healing rates in the OFM group [40].

The regenerative properties of OFM have also been extended to the management of complex volumetric defects in surgical settings, including contaminated environments frequently encountered in orthoplastic and limb salvage procedures. Cormican et al. [41] reported a clinical case series in which OFM, applied both as a graft and in particulate form, was successfully used to promote granulation tissue formation in large soft tissue defects with exposed internal structures (e.g., tendons, bone, viscera), even in patients with significant comorbidities. Complete granulation tissue coverage was achieved in a mean of 23.4 days, with no postoperative infectious complications [41].

In the field of orthopedic reconstructive surgery, OFM is emerging as a promising alternative for limb preservation in patients with chronic complex lower-extremity defects. Indeed, the management of postoperative soft-tissue defects in the lower legs presents a significant challenge due to arterial and venous insufficiency, poor skin quality (epidermal and dermal atrophy, reduced tissue elasticity), and an increased risk of infection. A prospective study by Lawlor et al. [42] followed 130 patients undergoing lower limb reconstruction using OFM grafts. Despite the high complexity of the cases, no major amputations or postoperative infections were reported. At 180 days, 62% of wounds were fully healed [42]. The study by Bosque et al. further supports the use of OFM as a clinically effective treatment for coverage of complex soft-tissue wounds [43].

These findings highlight OFM as a viable option to promote healing in critical scenarios where conventional treatments often fail. Its application in orthopedic and trauma surgery may potentially extend to implantology, both to protect exposed implants and to support the regeneration of adjacent soft tissues.

In orthopedic and implant-related contexts, in which regeneration of both bone and soft tissue is critical for successful surgical outcomes, decellularized matrices are particularly relevant [44]. In contrast to the OFM, mainly used for soft tissue reconstruction, the ovine decellularized matrix (ODM), derived from bone or dermis, has primarily been investigated for orthopedic bone regeneration. Produced through decellularization processes that preserve native 3D architecture and biochemical components of the ECM, ODM seems capable of supporting the regeneration of bone and connective tissues while maintaining high biocompatibility and a favorable immunological profile [45].

This bioscaffold is often combined with natural biomaterials such as chitosan, whose previously mentioned antibacterial and pro-healing properties enhance regeneration and help prevent post-implantation infections. The synergy between ODM and chitosan could be exploited to enhance osteogenesis and improve tissue integration, as demonstrated with other decellularized matrix–chitosan models [46,47].

In implantology, scaffolds with properties similar to ODM can be useful to promote the osseointegration of metallic implants and support bone remodeling. Functionalized scaffolds, potentially enriched with growth factors or antimicrobial nanoparticles (see subsequent paragraphs), represent an innovative strategy for optimizing clinical outcomes [48].

Overall, the combined use of biological matrices such as OFM and natural materials like chitosan can represent a multifunctional, synergistic strategy for tissue regeneration in orthopedic and implant-related surgery, enhancing healing quality while reducing infection-related risks.

### 4.3. Hydrogels and Dressings Containing Platelet-Derived Growth Factors: A Biological Support for Tissue Repair

The use of platelet-derived growth factors (PDGFs), already introduced in clinical practice, is still evolving for the treatment of challenging chronic wounds, such as diabetic ulcers and deep full-thickness wounds. Platelets are a natural source of pro-reparative molecules, including growth factors and chemoattractants, that stimulate neovascularization, cellular recruitment and proliferation, and extracellular matrix synthesis.

One of the most promising approaches involves platelet-based hydrogels (obtained from expired blood components originally intended for transfusion). In an experimental murine model, Rahman et al. [49] developed a platelet- and fibrin-rich hydrogel containing physiological concentrations of TGF-β1, PDGF-AB, PDGF-BB, IGF-1, FGF-2, and EGF. Applied to full-thickness wounds, this hydrogel significantly enhanced vascularization in the wound bed [49]. The authors hypothesize that the sustained and localized presence of IL-6 they also observed may directly contribute to the angiogenic stimulation. In this context, it is worth noting that, although primarily known as a pro-inflammatory cytokine whose elevated levels are considered pathological markers [50], IL-6 also plays crucial physiological roles in reparative and angiogenic processes [51] and exhibits complex pleiotropic functions.

A further advancement is represented by the design of bilayer dressings that enable the controlled and sustained release of platelet-derived factors. In a recent study, Alizadeh et al. [52] developed a bilayer dressing composed of a sodium tripolyphosphate-crosslinked gelatin sponge (Gel-STPP) layer and a top layer of carrageenan nanofibers containing platelet-rich fibrin (PRF). This bilayer demonstrated gradual release of PDGF and VEGF for at least seven days, significant improvement in mechanical properties compared to single layers, and excellent capacity to support the adhesion and proliferation of L929 cells. In vivo, the dressing achieved an average wound closure of 94.21% after 14 days, with the formation of a well-structured new epidermal layer [52].

Interest in these blood-derived biomaterials stems not only from their natural biocompatibility but also from the possibility of recycling blood components no longer suitable for transfusion, thus offering a sustainable, biologically active, and readily available therapeutic option. The integration of these systems into clinical routines could represent a step forward in managing chronic wounds, especially in patients with complex vascular and regenerative deficits such as those with diabetes.

In conclusion, hydrogels and dressings containing PDGFs can represent a biological support for tissue repair, with a potentially significant impact in orthopedic surgery and implantology. Their application could therefore improve outcomes in complex procedures, such as peri-implant tissue reconstructions and the management of surgical wounds in patients with comorbidities.

While platelets are a rich and accessible source of regenerative growth factors, several other cell types also contribute bioactive molecules that deserve consideration as key players in wound healing, vascularization, and bone repair.

Vascular Endothelial Growth Factor (VEGF) is a central mediator of angiogenesis, essential to vascularization and nutrient supply in regenerating tissues.

Not only platelets, but also endothelial cells, fibroblasts, macrophages, and keratinocytes, especially under hypoxic or inflammatory conditions, can secrete VEGF.

Transforming Growth Factor-β (TGF-β) and Platelet-Derived Growth Factor (PDGF), although present in platelet granules, are also actively produced by macrophages, fibroblasts, and mesenchymal stromal cells; TGF-β and PDGF are involved in both soft and hard tissue repair by regulating cellular recruitment, proliferation, and extracellular matrix remodeling.

Among the bone-specific regulators, Bone Morphogenetic Proteins (BMP-2 and BMP-7) stand out as potent osteo-inductive agents. They are secreted by osteoblasts, chondrocytes, and mesenchymal cells in response to injury. Recombinant human BMP-2 (rhBMP-2) and BMP-7 (rhBMP-7) have been introduced in clinical practice for selected orthopedic applications, such as spinal fusion and treatment of non-union fractures [53,54,55]. The integration of these growth factors into advanced biomaterial-based delivery systems, either alone or in synergistic combination, was initially thought to be a very promising strategy to enhance regenerative outcomes, particularly in complex wound scenarios involving both soft tissue and bone. Nonetheless, with time adverse effects such as soft-tissue inflammation, ectopic bone formation and tumor formation have emerged with the use of BMP-2, imposing caution and high control on dosing this bioactive molecule in bone regeneration. After an initial approval in 2001, a collagen-based product containing BMP-7 was withdrawn worldwide off the market in 2014 [56]. The supraphysiological dosages required to achieve adequate biological activity and their associated side effects have significantly limited the indication of use of currently existing BMP products.

Recent studies have thus been directed to achieve favorable control over bone regeneration by finely tailoring drug delivery systems for the controlled release of low doses of bone morphogenetic proteins alone or in combination with synergistic/complementary factors, for instance, low-dose BMP-7 and FGF-2 [57], low-dose BMP-2/Mg2+ [58], FK506 and low-dose BMP-2 [59], BMSCs and low dose BMP-2/polyelectrolyte complex (PEC) carrier system [60], low-dose BMP-2/tenascin-c functionalized self-assembling peptide hydrogels [61], and an ultra-low dose BMP-2 (5 μg/implant)/supramolecular polymer-collagen microparticle slurry [62].

### 4.4. Glycosaminoglycan Mimetics in Regenerative Medicine

Heparan sulfates (HSs) are key glycosaminoglycans (GAGs) within the extracellular matrix (ECM) that play a crucial role in tissue homeostasis and repair by binding growth factors, protecting them from degradation, and stabilizing the signaling microenvironment.

In chronic wounds, especially those of diabetic or vascular origin, the ECM is often degraded, and the regulation of these bioactive molecules is thwarted.

Glycosaminoglycan mimetics, such as Regenerating Agents (RGTA), are synthetic polymers designed to replicate the structure and function of heparan sulfates. By mimicking HS, RGTAs protect endogenous growth factors and ECM components from enzymatic and oxidative degradation typical of chronic inflammatory environments. This protective action underpins ECM reconstruction, which in turn stimulates cellular migration and promotes tissue regeneration.

Noticeably, clinical evidence has supported the efficacy of RGTA-based therapies. For example, a study on patients with diabetic foot ulcers showed that RGTA treatment accelerated wound closure and improved the quality of newly formed tissue [59].

## 5. Strategies for Tissue Regeneration in Orthopedic Wound Healing

### 5.1. Innovative Carrier Systems for Controlled and Targeted Therapeutic Delivery in Regenerative Medicine

Optimizing the delivery of drugs and bioactive molecules is fundamental for achieving therapeutic efficacy in tissue regeneration while reducing side effects and dosages.

Carrier systems such as nanoparticles, hydrogels, and functionalized scaffolds enable the controlled, targeted, and sustained release of active agents.

Numerous studies have highlighted the effectiveness of chitosan -based carriers due to their biocompatibility, biodegradability, and ability to interact with various therapeutic molecules. For example, chitosan nanoparticles have been successfully used to deliver antibiotics, growth factors, and anti-inflammatory agents, improving their stability and local release at the injury site [63,64,65]. This delivery system offers several advantages for enhanced orthopedic wound healing by providing potential antimicrobial activity and sustained drug release, outperforming conventional carriers in infection control and bioavailability.

Beyond chitosan, more sophisticated carrier systems are under development, such as multifunctional hydrogels integrated with biomimetic nanoparticles that can release oxygen, reduce oxidative stress, and promote cellular regeneration. An innovative example in this regard is hydrogels containing polydopamine-coated manganese dioxide nanoparticles, which act as nanozymes to degrade reactive oxygen species and generate oxygen, enhancing diabetic wound healing. These nanoparticles could add further relevance to hydrogel-based strategies, previously detailed, by facilitating targeted drug delivery and strong adhesion to tissue surfaces, enhancing therapeutic efficacy and retention over other delivery strategies [36].

Another significant development concerns the encapsulation of growth factors within carrier scaffolds to ensure sustained and localized release, directly stimulating regenerative processes such as angiogenesis and extracellular matrix formation [57,66,67,68].

Across clinical settings, from orthopedics to advanced wound care, multifunctional carrier systems provide a valuable approach to personalized regenerative therapies, minimizing infectious and inflammatory complications.

### 5.2. Advanced Biomaterials for Dermal Regeneration and Chronic Wound Management

Advanced biomaterials for dermal regeneration represent a natural evolution of strategies based on glycosaminoglycan mimetics and carrier systems for the controlled delivery of bioactive molecules. Specifically, scaffolds functionalized with RGTA and smart hydrogels offer new perspectives for managing chronic wounds and preventing fibrosis, thus improving the quality of skin regeneration.

Fibrosis development after full-thickness wound healing, especially after severe burns, remains a significant clinical challenge. Gansevoort et al. [69,70] developed a porous type I collagen scaffold covalently functionalized with a heparan sulfate mimetic, capable of capturing fibroblast growth factor 2 (FGF-2), known for its inhibitory effect on myofibroblasts responsible for fibrosis. Compared to scaffolds functionalized with heparin, this collagen scaffold exhibited sustained release of FGF-2 and in vitro anti-fibrotic potential, reducing the gene expression of myofibroblast and fibrosis markers even in the absence of FGF-2, thus demonstrating an intrinsic ability to enhance skin regeneration by limiting excessive scarring [69,70]. Hemalatha et al. [71] developed a hybrid hydrogel composed of natural silk fibroin and recombinant collagen-like proteins with binding domains. The riboflavin- and visible-light-activated crosslinking system enables the formation of a stable gel with improved physicochemical properties (elastic modulus and thermal stability) compared to silk fibroin controls. This hybrid hydrogel was shown to effectively support cell adhesion, elongation, proliferation, and migration in vitro, promote wound closure in cellular models, and modulate the expression of cytokines and growth factors [71]. Gao et al. [72] developed a redox-responsive hyaluronic acid (HA)-based hydrogel sensitive to glutathione (GSH) concentration variations typical of healing or non-healing wounds. Using a GSH-sensitive crosslinker, the hydrogel changes its structure in response to the metabolite level, potentially allowing low-cost visual diagnosis of wound status. Furthermore, the hydrogel demonstrated biocompatibility and supported fibroblast growth and proliferation, suggesting dual use as both a diagnostic device and an implant for tissue regeneration [72]. This latest application introduces the following section, focused on stimuli-responsive (nano)biomaterials.

In the context of advanced biomaterials, another recently emerged strategy consisting of stimuli-responsive nanoscaffolds has emerged as a promising strategy for the treatment of chronic wounds, particularly those associated with diabetes. These smart materials can respond to specific environmental stimuli, modulating the release of bioactive agents and regulating oxidative stress, two key factors in diabetic wound healing. Notable examples include cerium oxide nanoparticles, Prussian blue hydrogels, and graphene oxide-metformin hybrids [73,74,75].

Cerium oxide is well known for its antioxidant properties, enabling it to neutralize free radicals responsible for tissue damage. Moreover, recent studies demonstrate its role in promoting tissue regeneration, particularly in bone healing, by modulating inflammation, enhancing osteogenic differentiation, and creating a favorable environment for repair. In a recent study by Wu et al. [76], incorporation of cerium oxide nanoparticles into the micro-arc oxidation layer promoted bone formation and ensured structural integrity in magnesium orthopedic implants. This approach mitigated stress-shielding (a phenomenon in which the implant absorbs most of the mechanical load, diverting it away from the bone and thus hindering bone regeneration and implant integration) while stimulating biological processes essential for healing. Cerium oxide nanoparticles enhanced the biodegradability of magnesium and significantly facilitated bone regeneration [76].

Similarly, Prussian blue hydrogels provide controlled release of therapeutic molecules while dampening local oxidative stress. Particularly innovative are the graphene oxide-metformin hybrids, which combine the physicochemical properties of graphene with the pharmacological efficacy of metformin to enhance tissue regeneration.

The ability to modulate oxidative stress and release bioactives makes these materials especially suitable for the complex environment of diabetic wounds [77].

Furthermore, the use of hybrid nanoparticles loaded with curcumin represents a new potential tool in combating diabetic wound complications. These multifunctional systems not only counteract oxidative stress but also modulate inflammation and fight infection, thus favoring healing [78].

Stimuli-responsive nanoscaffolds represent an advanced frontier in regenerative medicine, combining targeted therapeutic capabilities with controlled release mechanisms.

The discussion of curcumin-loaded nanoparticles leads to the next section, which focuses on (nano)biomaterials enriched with natural substances.

### 5.3. Natural Extract-Loaded Nanofibers and Nanoparticles

Nanofibers show promise for wound healing by supporting active molecule delivery and tissue regeneration [79].

Electrospinning is a versatile and widely used technique to produce nanofibrous scaffolds characterized by high porosity and large surface area, which closely mimic the extracellular matrix. This method allows for the incorporation of natural extracts and therapeutic agents into the fibers, enabling sustained and controlled release directly at the wound site, thus enhancing tissue regeneration.

Electrospun nanofibers loaded with natural extracts and therapeutic agents represent an innovative approach for chronic wound treatment [80]. As previously noted, effective management of skin wounds remains a true clinical challenge due to bacterial infections, dysfunctional fibroblast activity, poor angiogenesis, and altered tissue remodeling. In this context, electrospun scaffolds incorporating natural extracts such as curcumin, metformin, and plant-derived substances like aloe vera, fenugreek, and calendula have demonstrated significant anti-inflammatory and pro-angiogenic activities in preclinical models. A recent approach explores the use of curcumin-cyclodextrin hybrid nanoparticles (Cur/CD-HNPs) as a multifunctional system to support wound healing. These nanoparticles, obtained through nanoprecipitation and thoroughly characterized, have shown notable anti-inflammatory activity by inhibiting protein denaturation, along with antioxidant and broad-spectrum antibacterial properties [78]. The curcumin release profile from the nanoparticles follows a sustained biphasic pattern, with 82% released over 24 h, supporting prolonged administration suitable for wound healing applications.

In vitro scratch assays further demonstrate that Cur/CD-HNPs promote cell proliferation and migration. In vivo, when incorporated into a hydrogel and applied locally to a rat burn wound model, Cur/CD-HNPs significantly accelerate wound closure. Histopathological analysis reveals improved epithelialization, collagen deposition, and tissue regeneration compared to controls. These findings suggest that Cur/CD-HNPs are able to ameliorate curcumin’s bioactivity, stability, and regenerative potential, overcoming major limitations of curcumin and constituting a promising multifunctional platform for wound treatment [78].

### 5.4. Silk Fibroin-Based Platforms, DOPA-Inspired Adhesives and Nanodiamond–Silk Fibroin Composites

Silk fibroin (SF)-based platforms represent an emerging class of biomaterials for tissue regeneration due to their mechanical properties, biocompatibility, and ability to be modified with bioactive molecules such as dopamine or berberine to regulate cytokine expression and promote epithelial and vascular tissue regeneration. Sang et al. developed sprayable berberine-silk fibroin microspheres with an extracellular matrix anchoring function, capable of continuously releasing berberine and exerting antibacterial and anti-inflammatory effects even in the presence of abundant exudate, accelerating the healing of infected wounds [81]. Similarly, Sha et al. [82] developed a biomimetic scaffold based on a hydrogel composed of hyaluronic acid, silk fibroin, and polydopamine for the prolonged release of neurotrophin-3, a crucial factor for axonal regeneration in the spinal cord. This system demonstrated an attenuation in lesion cavity formation and inflammation, improving hind limb motor function in experimental models [82].

Another advancement involves integrating regenerated silk fibroin (RSF)-coated liquid metal nanoparticles into photothermal hydrogels with antibacterial properties enhanced by near-infrared (NIR) irradiation. This system promotes the healing of infected wounds by facilitating epithelialization and organized collagen deposition, achieving complete skin and hair recovery within 14 days post-injury [83]. Adhesive properties inspired by catecholamines, typical of DOPA-inspired adhesives, offer an innovative approach for wound closure in moist environments, providing strong adhesion, biocompatibility, self-healing, and controlled drug release capabilities. These hydrogels mimic mussel adhesive mechanisms, allowing not only the closure of internal and external wounds but also antimicrobial properties and promotion of hemostasis.

Finally, nanodiamond–silk fibroin composites represent an innovative multifunctional platform for wound monitoring and healing. These hybrids, produced by electrospinning, form porous fibrous membranes capable of acting as optical temperature sensors, a key biomarker for detecting infection or inflammation without removing the dressing. The incorporated nanodiamonds improve the thermal stability of the fibroin while maintaining fluorescence and magnetic resonance optical properties for temperature detection within the relevant biological range (25–50 °C). In murine models, these membranes demonstrated biocompatibility, effectiveness in wound closure, and selective antibacterial activity against Gram-negative bacteria such as *Pseudomonas aeruginosa* and *Escherichia coli*, without, however, affecting *Staphylococcus aureus* [84].

Silk fibroin-based platforms enriched with bioactive molecules, functional nanomaterials, and nature-inspired adhesive systems combine regenerative capacity, real-time monitoring, and offer antibacterial protection to varying degrees.

### 5.5. Functionalized Clay Membranes

Functionalized clay-based membranes represent a sustainable solution for the treatment of infected wounds, thanks to their broad antimicrobial efficacy and suitability for large-scale production. Clay, known for its natural therapeutic properties and abundance, is a highly promising biomedical material due to its non-toxic nature, high porosity, large specific surface area, and cation exchange capacity. Recent developments have led to the creation of clay hybrids enriched with silver ions and antifungal agents such as terbinafine, which exhibit broad-spectrum antimicrobial activity. In particular, the functionalization of these membranes also involves the use of zwitterions, which further enhance microbial resistance. These materials have been tested against common skin wound pathogens such as *Staphylococcus aureus*, *Escherichia coli*. The study by Ghimire et al. [85] demonstrated an innovative approach to engineering an infection-resistant bandage material from clay. The hybrid membranes were developed using clays, zwitterions, silver ions, and terbinafine hydrochloride (TBH) to provide antibacterial and antifungal efficacy. Results demonstrated the potential of these hybrid clay membranes as a cost-effective, easily producible on a large scale, and effective solution for treating microbial infections [86,87,88,89]. In conclusion, these hybrid membranes are a promising therapeutic platform for managing infected wounds, particularly in resource-limited settings.

### 5.6. Bioactive Agents Revisited Through Advanced Delivery Systems

This paragraph concludes by spotlighting how some well-known biomolecules and bioactive agents are being revisited through their incorporation into innovative delivery systems or advanced (nano)formulations. Fibronectin, a major ECM protein, has recently been revisited through its combination with lysostaphin in silk fibroin dressings [90] and its application in extracellular matrix spray-coating strategies for surface functionalization and tissue repair [91]. Elastin, another extracellular matrix (ECM) protein, has been investigated in sponge form for the treatment of skin defects in a phase III clinical trial [92]; in addition, elastin-like polypeptides have been proposed as grafting platforms for regenerative scaffolds [93]. Heparin, previously recognized for its ability to inhibit bacterial adhesion when employed as a biomaterial coating [94], is now being repurposed for wound-healing applications via spray-based formulations [95], peptide reservoirs [96], and patch-based delivery systems [97]. Medical-grade honey is attracting renewed interest and is currently incorporated into a wide range of formulations, including dressings [98], gels [99], sponges [100], and nanocoated membranes [101]. Finally, silver-based wound dressings, used for decades, are being critically re-evaluated to better define their role in the management of infected wounds and in the prevention of surgical site infections, while novel formulations based on silver nanoparticles are being actively explored [102,103].

## 6. Peptides-Based Strategies for Wound Healing

### 6.1. GHK-Cu Peptides and Their Regenerative Applications

The natural tripeptide GHK (Glycyl-L-Histidyl-L-Lysine), particularly in its copper-complexed form (GHK-Cu), is a compound naturally present in human plasma, saliva, and urine. It is known for its regenerative, anti-inflammatory, and antioxidant properties. GHK-Cu supports collagen production and angiogenesis while exerting antioxidant effects and reducing the expression of pro-inflammatory cytokines. When incorporated into modified silver nanoparticles, both GHK and GHK-Cu peptides have demonstrated effective antibacterial activity against *Staphylococcus aureus* and *Escherichia coli*, with minimum inhibitory concentrations (MICs) of 8 μg/mL. In infected in vivo models, these systems accelerated cell migration and enhanced wound healing, as evidenced by increased epidermal thickness, greater collagen deposition, and downregulation of TNF-α expression [104]. The use of a reactive oxygen species (ROS)-responsive hydrogel incorporating a dimeric copper peptide (D-CuP) demonstrated greater biological and chemical stability compared to the monomeric form, with targeted and sustained peptide release at the wound site. This intelligent system, combining ROS scavenging capacity and inflammation modulation, achieved 97.2% closure of infected wounds in diabetic models, highlighting its therapeutic potential for difficult-to-treat chronic wounds [105]. These findings position GHK-Cu peptides and their advanced formulations as promising multifunctional biomaterials in regenerative medicine for wound treatment, offering synergistic antibacterial, anti-inflammatory, and tissue-regenerating effects.

### 6.2. Antimicrobial Peptides in Wound Healing: Infection Control and Tissue Regeneration

Antimicrobial peptides (AMPs) are short-chain molecules, typically composed of 10 to 50 amino acids. They constitute a crucial component of the innate immune system in numerous organisms and are produced by bacteria (bacteriocins) as part of their survival strategy against other microbes within animal microbiota. AMPs are active against both Gram-positive and Gram-negative bacteria, and many exhibit antifungal and antiviral properties. As antibacterial agents, they differ from traditional antibiotics in that they primarily act by disrupting microbial membranes, thus significantly reducing the risk of resistance development. Beyond their antimicrobial activity, certain AMPs directly contribute to the wound healing process. Among the many functions expressed by the different peptides, some modulate inflammation, stimulate cell migration and proliferation, and promote extracellular matrix deposition. This dual action of infection control and promotion of repair makes AMPs ideal candidates for therapeutic applications in chronic and complex wounds.

Among the most studied AMPs are LL-37, a human peptide belonging to the cathelicidin family, known for its ability to modulate immunity and promote tissue repair; defensins, cationic peptides involved in microbial membrane disruption and immune response regulation; and cathelicidins in general, which exhibit both direct antimicrobial effects and immunomodulatory properties. Synthetic or chemically modified peptides have been developed to enhance stability and mitigate toxicity. The clinical use of AMPs faces important challenges related to their limited stability and bioavailability in biological environments due to enzymatic degradation [106]. Various delivery strategies are currently under development to improve their therapeutic efficacy such as incorporation into biocompatible hydrogels for the controlled release and maintenance of a moist environment conducive to healing. Embedding into nanofibers and AMP-loaded films is useful for direct wound protection and prolonged action. Design of stimuli-responsive delivery systems, such as those sensitive to pH or specific enzymes, can release the peptide in response to biological signals from the wound microenvironment. Regarding orthopedic-aimed studies, the potential of AMPs has been demonstrated in terms of infection prevention rather than wound healing directly. AMP-loaded calcium phosphate cement outperformed antibiotic-loaded cement in eradicating infection and preventing biofilm formation on model implants in human femur tissue, demonstrating efficacy against osteomyelitis, a severe bone infection often associated with implant-related complications [107]. AMP-functionalized titanium implants achieved near-complete bacterial clearance while enhancing bone regeneration and wound repair in a rabbit infection model [108]. Of note, an engineered peptide, namely PLG0206, has reached Phase 1 (ClinicalTrials.gov ID: NCT05137314) to assess its safety for use in PJI, although conclusive data have not been reported yet. Despite their therapeutic potential, the clinical use of AMPs still raises concerns, including limited stability in biological fluids, possible toxicity or undesirable immune responses, high production costs compared to conventional antibiotics, and the need for broader clinical studies and well-defined regulatory frameworks. In the orthopedic context and in difficult wound scenarios, AMPs could represent a valuable tool for preventing post-operative infections and accelerating bone and soft tissue regeneration. Further clinical trials and the development of synthetic AMPs with optimized pharmacological profiles will be essential to translate these promising molecules from experimental models into routine clinical practice.

## 7. Integration of Smart Technologies in Wound Management

In recent years, wound care has significantly advanced through the integration of smart technologies that combine advanced biomaterials, bioelectrical stimulation, and intelligent sensing systems. These innovations offer new approaches for real-time monitoring and personalized therapeutic delivery. One of the most promising examples is a water-powered, electronics-free dressing, a smart dressing capable of delivering localized electrical stimulation without the need for external power or electronics. In diabetic mouse models, these systems have been shown to accelerate wound closure, increase epidermal thickness, stimulate angiogenesis, and relieve local inflammation [109].

At the same time, biodegradable bioelectric surgical sutures exploit the triboelectric effect to deliver continuous, localized electrical stimulation during wound closure. These self-powered materials do not require external devices and actively promote tissue regeneration while acting as a barrier against infections [110,111]. Lastly, smart sensory bandages represent a technological leap toward dynamic wound monitoring and personalized care. These flexible dressings integrate miniature sensors that detect local parameters, such as pH, moisture, and temperature, frequently associated with infection and inflammation. They may include systems for controlled drug delivery and antibacterial treatment via colorimetric, electrochemical, photothermal, or electrical stimulation methods [112]. These smart technologies represent a new generation of tools in regenerative medicine, offering more effective, less invasive, and customizable wound management strategies, particularly impactful in the treatment of chronic wounds and in resource-limited settings.

## 8. Other Significant Emerging Strategies

In addition to the emerging biomaterial-based approaches discussed, several other therapeutic strategies are under active investigation, though currently characterized by limited clinical translation or incomplete mechanistic understanding.

Mesenchymal stem cells (MSCs), particularly those derived from bone marrow, adipose tissue, and placental tissues, are being extensively explored for their regenerative and immunomodulatory potential in wound healing and tissue repair [113,114]. MSCs secrete a wide array of bioactive molecules, including growth factors, cytokines, and extracellular vesicles, that play critical roles in modulating inflammation, promoting angiogenesis, and enhancing extracellular matrix (ECM) remodeling [113,114]. A key determinant of the therapeutic efficacy of MSCs lies in the source tissue from which they derive. The tissue of origin significantly influences the phenotypic and functional characteristics of MSCs, affecting their regenerative capacity, immunomodulatory properties, and secretory profiles. As such, selecting the optimal MSC source is essential to tailoring effective, personalized therapeutic strategies [114]. Among the emerging alternatives to MSCs alone, or as supportive platforms for MSC-based therapies, placental-derived biomaterials have garnered significant attention due to their unique biological advantages [115]. These include biocompatibility, pro-angiogenic, anti-inflammatory, antimicrobial, antifibrotic, and immune-privileged properties. Even more importantly, the ECM of placental tissues serves as a natural scaffold that supports cell migration, proliferation, and differentiation while also contributing to ECM remodeling essential for effective wound repair [113]. Placental-derived scaffolds have demonstrated promising results in preclinical studies, supporting their safety and efficacy in enhancing wound healing. However, the current literature indicates that differences among placental anatomical regions and their specific contributions to regenerative outcomes remain underexplored. Furthermore, despite encouraging results, challenges such as cell survival, engraftment, and regulatory hurdles continue to limit the widespread clinical adoption of both MSC-based and placental-derived therapies. In conclusion, the success of regenerative approaches using MSCs and placental derivatives hinges on the comprehensive characterization of cell sources, scaffold properties, and the regenerative microenvironment. Future research should prioritize standardization of isolation methods, comparative studies across tissue sources, and long-term clinical evaluations to unlock the full therapeutic potential of these biologics [113].

Targeting immune pathways to optimize inflammation resolution and promote tissue repair is also gaining attention. Modulating macrophage polarization and cytokine profiles can enhance wound healing outcomes, but effective, specific therapies remain under development [116]. In this regard, an emerging advantageous therapeutic alternative comes from exosomes. These nanosized vesicles secreted by cells, particularly stem cells, mediate intercellular communication and carry bioactive cargo such as proteins and RNAs. Exosomes offer superior cellular therapy potential due to their natural biocompatibility, intrinsic targeting, efficient cargo delivery, immune-modulatory effects, and lower risk of tumorigenicity compared to other nanovesicles, thus setting the grounds for future precision-delivery systems. Their use as cell-free regenerative agents shows promise in modulating inflammation and promoting tissue repair but requires more extensive clinical validation [117].

Advanced 3D printing techniques aim to fabricate complex tissue scaffolds with precise spatial control of cells and bioactive molecules. While promising for customized wound dressings and tissue constructs, this technology requires further refinement for routine clinical use [118,119,120].

Gene editing and delivery of therapeutic microRNAs offer potential to correct underlying molecular defects in chronic wounds. Current research focuses on safe and efficient vectors for localized gene modulation, though clinical applications are still nascent [121,122]. CRISPR/Cas9 gene editing is a powerful tool to enhance the efficacy of cell-based therapies for wound healing. Recently, CRISPR-mediated knockout of Ndrg2 in dendritic cells (DCs) has been used to accelerate healing in diabetic and non-diabetic wounds. However, how genetically engineered cells interact with local cell types of the wound healing environment to accelerate healing is still unknown [123].

The evolving integration of bioinformatics in the clinical field has also brought attention to the development of AI-driven diagnostic tools and predictive models to optimize personalized treatment plans and monitor healing progression. Integration with smart dressings and imaging modalities is an exciting frontier, albeit in its early stages [124,125,126,127,128].

Furthermore, low-intensity ultrasound has demonstrated the ability to enhance tissue regeneration via the mechanical stimulation of cells, increased angiogenesis, and inflammation modulation. This non-invasive approach holds potential for chronic wound treatment, with ongoing clinical studies aimed at establishing efficacy and protocols [129,130,131].

These complementary strategies, while still in development or limited by technological and regulatory hurdles, represent a diverse and promising research landscape poised to enrich the future of wound healing and regenerative medicine. Together, they represent a broad frontier of ongoing research, with the potential to synergize with biomaterial-based therapies and smart technologies, ultimately advancing the effectiveness and precision of regenerative medicine (see Table 1).

## 9. Articular Cartilage Regeneration: An Unresolved Challenge

Articular cartilage, due to its avascular, aneural, and alymphatic nature, has minimal intrinsic regenerative capacity. Its complex zonal architecture and specialized extracellular matrix-rich in type II collagen and aggrecan pose significant challenges for effective repair. Conventional strategies often fall short in reproducing its native biomechanics and long-term functionality. Current approaches aim to recreate the cartilage microenvironment through stem cell-laden scaffolds, growth factor delivery, gene therapy, and 3D bioprinting. However, critical hurdles persist, including achieving stable integration with subchondral bone, restoring tissue anisotropy, and avoiding the formation of fibrocartilage. Emerging frontiers involve bioactive hydrogels with spatially controlled cues, multizonal scaffolds mimicking depth-dependent architecture, and patient-specific constructs. While these strategies show promise, their clinical translation remains limited. An excellent and up-to-date review by Pueyo Moliner et al. provides an in-depth analysis of the structural, compositional, and functional aspects of articular cartilage, alongside biological mechanisms and regenerative strategies [132]. Similarly, Novotná and Franková presented a focused review on functional biomaterials for osteochondral regeneration, highlighting the relevance of multifunctional platforms in addressing joint complexity [133]. Lin et al. contribute an advanced study on biomimetic multizonal scaffolds capable of reconstructing the stratified organization of cartilage in both chondral and osteochondral defects [134], offering innovative insights into joint tissue engineering.

**Figure 3:**
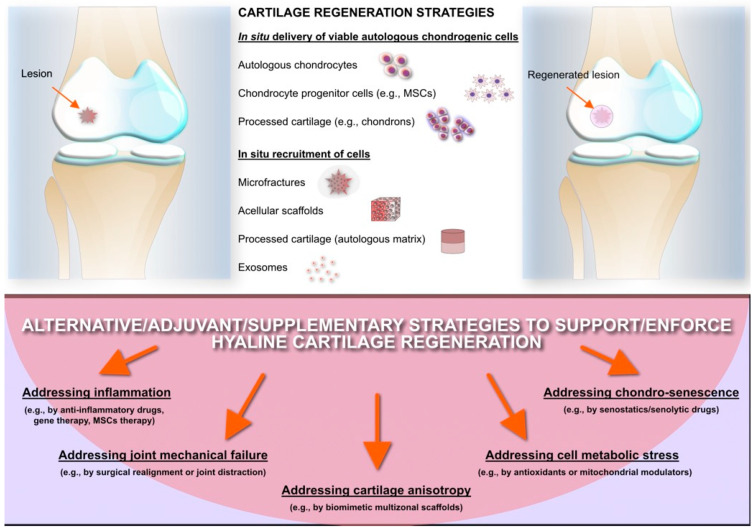
Current strategies that have been attempted and are being pursued to repair large lesions of knee joint cartilage.

Most current approaches to repair cartilage are uniquely attempting to repopulate the lesioned cartilage with chondrogenic cells characterized by a stable phenotype and are capable of generating perfectly functional hyaline cartilage and, thus, finally restore fine mechanical functionalities of the native, highly performing, healthy tissue. However, apart from artificially recreating the perfect matrix, with the same architecture both in terms of molecular composition and ultrastructure, a more holistic approach should be attempted, considering all pathological mechanisms that cause otherwise healthy cartilage tissue to become damaged. A very interesting review by Muthu et al. [135] recently enlisted a series of different hypotheses for why articular cartilage regeneration fails, which, to a certain extent, corresponds to why initially healthy cartilage degenerates. Probably a more comprehensive strategy to address the regeneration of articular cartilage should take into consideration all the possible factors determining cartilage damage, among those identified by Muthu et al. [135]: cell senescence, inflammatory stress, metabolic stress, and mechanical failure. We could further hypothesize the role of undetected infections where bacteria survive intracellularly in chondrocytes, a possibility that is emerging from recent in vitro studies [136], similarly to what happens in osteoblasts [137]. In the upper part of Figure 3, a series of cartilage regeneration strategies are reported, these aim at the delivery of viable cells with chondrogenic potential (either differentiated chondrocytes or progenitor cells) or at their local recruitment at the lesioned site. In the lower part of the same figure are conversely enlisted different approaches that are currently being attempted to address single specific causes of cartilage repair failure (likely, also the cause of chondrocyte damage and cartilage degeneration) but that could probably be combined in a more articulated and comprehensive strategy to ensure greater chances of success in functional cartilage regeneration.

## 10. Conclusions

This review highlighted emerging multifunctional strategies and advanced biomaterials designed to address the complex pathophysiology of wound healing, with particular attention to orthopedic and implant-associated wounds. From silk fibroin-based systems and copper peptides to antimicrobial membranes and smart dressings, current approaches converge on platforms that integrate antimicrobial protection, immune modulation, and regenerative stimulation.

Despite remarkable scientific progress, the leap to clinical translation remains hindered by major obstacles. Foremost among these is the challenge of achieving industrial-scale production of multifunctional therapies, a barrier made even more daunting by the prohibitively high current costs of the technologies involved. Additional constraints stem from incomplete ethical and regulatory frameworks, though these are expected to evolve as the technologies advance.

Moreover, significant biological barriers persist, particularly those related to the development of customizable, integrated approaches that can effectively traverse physiological constraints and accommodate patient-specific variability, thereby limiting their readiness for clinical implementation.

Future progress will need to address these issues and develop adaptive, personalized, and bio-responsive systems, supported by technologies like gene editing, 3D bioprinting, and AI-guided design. Noticeably, the organ-on-chip technology is gaining momentum as an innovative and promising tool, offering unprecedented precision in modeling wound healing and filling the gap between traditional in vitro studies, functional biomaterials, and human physiology.

Bridging basic science, bioengineering and clinical application, especially in complex wound scenarios, will require strong interdisciplinary collaboration to transform experimental platforms into effective, next-generation therapies.

## Figures and Tables

**Table 1:** Summary view of the main strategies under development for orthopedic wound healing.

Therapeutic Strategy	Key Advantages and Disadvantages	Primary Clinical Applications
**Advanced Bio-Scaffolds** (*Chitosan*, *Silk Fibroin*, *OFM*, *ODM*, *3D Bioprinting*)	**Structural and environmental support:** Provides native 3D architecture (osteoconduction for ODM), mimics ECM (OFM) and enables precise spatial control of cells; but high cost, complex fabrication, potential immunogenicity.	Soft tissue reconstruction, bone defects, limb salvage, tendon exposure (OFM), bone regeneration (ODM), and complex anatomical reconstruction.
**Bioactive Factors and Peptides** (*PDGFs*, *BMPs*, *GHK-Cu*, *AMPs*, *GAG Mimetics*)	**Signaling and protection:** stimulation of angiogenesis and matrix synthesis, antimicrobial activity (membrane disruption), redox modulation, and protection of growth factors from enzymatic degradation; but short half-life, stability issues, risk of off-target effects.	Non-union fractures, infected biofilm-associated wounds, and chronic diabetic ulcers.
**Cellular and Gene Therapies** (*MSCs*, *Placental derivatives*, *Exosomes*, *CRISPR*)	**Paracrine and genetic modulation:** Secretion of regenerative cargo (EVs, cytokines), immunomodulation (macrophage polarization), and targeted correction of molecular defects; but high cost, potential tumorigenicity or immune reactions.	Complex regenerative deficits, immune-privileged healing contexts, and personalized regenerative strategies.
**Smart Technologies and Nanomaterials** (*Sensors*, *Bioelectric Dressings*, *Cerium/MnO_2_ NPs*)	**Responsive monitoring and stimulation:** Real-time sensing (pH/Temp), electrical stimulation of repair processes and stimuli-responsive antioxidant activity; but complex design, potential cytotoxicity, scalability challenges.	Diabetic foot management, theranostic (therapy + diagnostic) approaches, and oxidative stress reduction in chronic wounds.
**Inorganic & Hybrid Systems** (*Functionalized Clays*, *Ag/Curcumin-loaded Nanofibers*)	**Delivery & Stability:** High absorption capacity, sustained release of antimicrobial ions or natural compounds, and broad-spectrum infection control; but potential cytotoxicity, limited biodegradability, burst release risk.	Infected wounds in resource-limited settings and in the management of highly exudative wounds.

## Data Availability

Data conceptualization is contained within the article.
